# Supplementary material for: Social avoidance can be quantified as navigation in abstract social space
Source: Commun Psychol. 2025 Mar 25;3:51. doi: 10.1038/s44271-025-00215-8 (PMC11936828; doi:10.1038/s44271-025-00215-8)
Supplement: Supplementary file 2 — Supplementary Information [file 44271_2025_215_MOESM2_ESM.pdf]

Supplementary Information

Supplementary Note 1: Questionnaire summary statistics

The questionnaire set was designed to capture broad variation in social avoidance, mood and compulsion. In supplementary table 1, we provide the summary statistics of the different questionnaires.

Questionnaire (construct)	Initial sample mean (SD)	Validation sample mean (SD)
Zung Self-Rating Depression Scale (Depression)	38.3 (11)	41.3 (11.1)
Apathy Evaluation Scale (Apathy)	36.8 (12.7)	38.1 (12.4)
Liebowitz Social Anxiety Scale avoidance subscale (Social Anxiety)	32.4 (16.1)	34.8 (16)
Avoidant Personality Disorder Impairment Scale (Avoidant Personality)	9.9 (6.5)	10.2 (6.9)
Short Scales for Measuring Schizotypy (Schizotypy)	12.5 (7.7)	12.9 (7.8)
Broad Autism Phenotype Questionnaire (Autism)	3.1 (0.7)	3.2 (0.7)
Zanarini Borderline Personality Disorder (Borderline Personality)	0.32 (0.3)	0.33 (0.3)
Obsessive Compulsive Inventory-Revised (Compulsion)	12 (11.6)	16 (13.4)

Supplementary table 1. Summary statistics of questionnaires. Means and standard deviations of the different self-report questionnaires in the two different samples (Initial sample n = 579, Validation sample n = 255).

Supplementary Note 2: Factor analysis regressions are more interpretable than questionnaire- and item-level regressions

To validate our assumptions for using factor analysis, we also ran regression models with the questionnaires themselves. We regressed the task first-person social distance onto models for each questionnaire score and item and models combining all questionnaire scores and items. We expected the factor analysis to have stronger and more replicable effects, and to be more interpretable.

Regressions with questionnaire scores in separate models yielded similar findings across the two samples. The two samples' top five questionnaires (judged by β values) overlapped, but with different ranks: the Initial sample's top five were Autism, Apathy, Avoidant Personality, Schizotypy and Depression, while the Validation sample's top five were Social Anxiety, Apathy, Avoidant Personality, Autism and Schizotypy. This suggests a large role for apathy–but this is likely because of social items in the Apathy questionnaire, which loaded onto the Social Avoidance factor.

Regressions with the questionnaire scores in one model provides less clear results: significant relationships for the Initial sample included the Borderline Personality and Apathy questionnaires, whereas significant relationships for the Validation sample included the Social Anxiety and Apathy questionnaires. Apathy was the only one common to both–creating the possible conclusion that the strongest and most replicable self-reported symptom relationship with social distance is mood. Our factor analysis regressions, as well as the social network analyses, clearly argue against this interpretation: while (non-social) mood has a small relationship with the behavior in the task, social self-reports have a much larger and more consistent effect.

Regressions with all questionnaire items in one model reveals how the score-level effects can obscure the actual underlying trait being studied. In both the Initial and Validation samples, several of the Apathy items are in the top 10 strongest effects: several of them relate to non-social apathy whereas the strongest item in both samples

is social in nature. The other items across the samples don't align. When the items are combined into a single model, effects are less statistically significant (again, reflecting the extensive correlations between items) and many don't survive multiple comparison correction or replication. As such, the picture is much less clear when using the questionnaires natively, either as summary scores or individual items.

Supplementary Note 3: The effects of affiliation and power are across characters

To test that the effects of affiliation and power on Social Avoidance are present across characters, we tested for character-level effects. We ran ordinary least squares regressions separately for each character, where the affiliation and power locations (along with standard controls) predicted Social Avoidance. The character-level effects were all consistent with the overall effect, with both affiliation and power showing negative relationships with Social Avoidance (all βs < 0; see **Supplementary table 2**).

Character ID	dimension	beta	95% C.I.	t-statistic	Left-tailed p-value
1	affiliation	-0.18	[-0.25, -0.11]	-5.21	< 0.001
1	power	-0.05	[-0.11, 0.02]	-1.35	= 0.089
2	affiliation	-0.11	[-0.18, -0.04]	-3.16	< 0.001
2	power	-0.06	[-0.13, 0.01]	-1.69	= 0.0452
3	affiliation	-0.11	[-0.17, -0.04]	-3.07	= 0.0011
3	power	-0.14	[-0.21, -0.08]	-4.17	< 0.001
4	affiliation	-0.15	[-0.15, -0.22]	-4.35	< 0.001
4	power	-0.10	[-0.17, -0.03]	-2.84	= 0.0023
5	affiliation	-0.06	[-0.13, 0.005]	-1.83	= 0.0337
5	power	-0.07	[-0.14, 0.001]	-1.99	= 0.0235

Supplementary table 2. Individual character regressions. We ran regressions for each character's affiliation and power location predicting Social Avoidance, to test if the effects were present across characters. Character ID is arbitrary. P-values are left-tailed, as this was a directional hypothesis.

Supplementary Note 4: First-person social distance explains Social Avoidance

Social interactions may be represented from a first-person point-of-view, where individuals represent others' locations relative to themselves in social space [29]. Consistent with this, previous research using this social navigation task found that neural representations are better explained by such a first-person framework, where relationships are represented as distances and angles from the participant's perspective (Tavares et al., 2015). In this framework, the orientation of the social vector indicates the interaction between power and affiliation relative to oneself, while the length represents absolute social distance, with greater distances reflecting lower affiliation and larger power differences.

We compared the ability of two coordinate systems (neutral and first-person) to explain the Social Avoidance effects, by calculating the distance and angle (r, θ) from a reference location for each affiliation and power location across the task. In the neutral coordinate system, these values were calculated from the origin coordinates (0, 0); angles were calculated as the counterclockwise angle between the vector from the origin and the positive affiliation axis:

$$r_{\text{neutral}} = \sqrt{\text{affiliation}^2 + \text{power}^2}$$

$$\theta_{\text{neutral}} = \text{arctan2}(\text{power}, \text{affiliation})$$

The angles were converted to the range [0, 360°] and transformed with the sine and cosine functions.

For the first-person coordinate system, distances were calculated from the maximum affiliation value and neutral power value (6,0); angles were measured between participant-to-character vector and the positive power axis [0, 180°] (see **Character relationships as affiliation and power trajectories** in the methods for formulas). We normalized the distances by z-scoring and the angles by the cosine transformation.

Previous work suggested that the first-person reference frame explained neural data better than the neutral reference frame; to test whether this is also the case for self-reported Social Avoidance, we compared the ability of these models to explain Social Avoidance scores. OLS-based BIC scores confirmed that the first-person model fit these scores better relative to the neutral model in both samples (as judged by smaller BIC; Initial sample: 1744.28 vs. 1761.93; Validation sample: 818.08 vs. 824.89). Some of this may be explained by parsimony: in the neutral representation, distance, sine angle and cosine angle are needed to fully specify each location, but in the first-person representation, only distance and cosine angle are needed to specify each location.

We then tested the first-person social distance and angle coefficients to compare their ability to explain Social Avoidance. Distance had a consistently significant relationship to Social Avoidance across both samples (Initial: $\beta = 0.21$, $CI_{95\%} = [0.13, 0.29]$, $t_{555} = 4.96$, right-tailed $P < 0.001$; Validation: $\beta = 0.25$, $CI_{95\%} = [0.12, 0.37]$, $t_{230} = 3.84$, right-tailed $P < 0.001$), whereas angle did not (Initial: $\beta = 0.11$, $CI_{95\%} = [0.03, 0.19]$, $t_{555} = 2.62$, right-tailed $P = 0.0045$; Validation: $\beta = 0.09$, $CI_{95\%} = [-0.04, 0.22]$, $t_{230} = 1.38$, right-tailed $P = 0.085$). These results suggest that while both social distance and social angle explain the Social Avoidance score, the social distance of the characters from the self is the best single predictor.

Supplementary Note 5: Self-reported character rating specificity analyses

After the task, participants (Validation sample only) rated the characters on a variety of social dimensions: likability and impact (as described in the main text, see **self-**

reported character ratings capture affiliation and power), as well as friendliness, popularity, competence and dominance. Each question was asked for each character, in a straightforward way (e.g., "How friendly was ___?"), in a random order, with a clickable slider from 1 to 100 to log the rating. Participants also rated themselves on these dimensions, for how they think people in the real-world perceive them.

In the main text, we show that, as predicted, likability and relative impact (the average difference between character impact ratings and the participant's rating of their own real-world impact) have specific relationships with affiliation and power, respectively. To gain greater insight into the affiliation and power behaviors, we ran additional exploratory analyses. We treated friendliness and popularity as alternatives to likability and related them to affiliation tendency. We calculated relative scores for competence and dominance (calculated as mean character rating minus self-rating) as alternatives to relative impact and related these to power tendency. We ran individual OLS regressions of each behavioral dimension (affiliation and power) with each of their rating dimensions and then compared BIC scores.

As expected, likability best fit the affiliation behavior (BICs: likability = 773.1, friendliness = 816.88, popularity = 820.05); testing the beta coefficients showed that friendliness had a significant effect (β = 0.13, $CI_{95\%}$ = [-0.002, 0.26], t_{232} = 1.92, right-tailed P = 0.027) but popularity did not (β = 0.06, $CI_{95\%}$ = [-0.07, 0.19], right-tailed P = 0.18). Also as expected, relative impact best fit the power behavior (BICs: relative impact = 800.21, dominance = 803.7, competence = 806.3); the beta coefficients for both relative dominance (β = 0.18, $CI_{95\%}$ = [0.06, 0.3], t_{232} = 2.92, right-tailed P = 0.0019) and relative competence (β = 0.15, $CI_{95\%}$ = [0.03, 0.27], t_{232} = 2.46, right-tailed P = 0.0073) were significant.

One possibility is that relative dominance and relative competence are features of power, and relative impact is a summary over both features. Consistent with this, when relative competence and dominance are used as predictors of power tendency, both are positive and significant (competence: β = 0.11, $CI_{95\%}$ = [-0.018, 0.24], t_{231} = 1.69, right-

tailed $P = 0.0462$; dominance: $\beta = 0.15$, $CI_{95\%} = [0.021, 0.27]$, $t_{231} = 2.28$, right-tailed $P < 0.0112$), but neither significantly relate to affiliation behavior (competence: $\beta = -0.06$, $CI_{95\%} = [-0.18, 0.07]$, $t_{231} = -0.87$, $P = 0.39$; dominance: $\beta = -0.02$, $CI_{95\%} = [-0.15, 0.1]$, $t_{231} = -0.34$, $P = 0.74$). These two rating dimensions also significantly relate to relative impact (relative competence: $\beta = 0.38$, $CI_{95\%} = [0.27, 0.49]$, $t_{231} = 6.8$, right-tailed $P < 0.001$; relative dominance: $\beta = 0.28$, $CI_{95\%} = [0.17, 0.39]$, $t_{231} = 5.1$, right-tailed $P < 0.001$), suggesting they may be different power-related features captured by relative impact.

Supplementary Note 6: Social Avoidance is related to social distance above and beyond likability and relative impact of characters

To test whether the Social Avoidance factor was related to behavior above and beyond the effects of character likability and relative impact, we also regressed the Social Avoidance scores onto the social distance measure, average likability, average relative impact as well as the standard controls. Even when controlling for these variables, social distance was significantly correlated with Social Avoidance ($\beta = 0.20$, $CI_{95\%} = [0.06, 0.33]$, $t_{230} = 3.84$, right-tailed $P = 0.0019$). This suggests that actual behavioral choices may be more closely linked to social avoidance than self-reported ratings.

Supplementary Note 7: Participants' perception of their own social standing correlates with self-reported Social Avoidance

We also tested whether the Validation sample participants' perceptions about their own real-world social standing related to their Social Avoidance factor scores. Participants rated how much they thought people liked interacting with them and how much impact they have over other people's goals, in the real-world, on a scale from 1-100. These two variables may be a proxy for the participants' perceptions of their own real-world social location (i.e., position along affiliation and power dimensions), given the relationships of

self-reported likability and impact with average affiliation and power task behavior, respectively (see main text section **self-reported character ratings capture affiliation and power**). Consistent with this, in OLS regressions affiliation was positively related to self-rated own likability (β = 0.19, $CI_{95\%}$ = [0.06, 0.32], t_{232} = 2.98, right-tailed P = 0.0016) and power was negatively related to self-rated own impact (β = -0.19, $CI_{95\%}$ = [-0.25, 0.003], t_{232} = -1.93, left-tailed P = 0.0274). We also expected these variables to negatively correlate with the Social Avoidance factor, and indeed, this is what we found (adj. R^2 = 0.21; own likability: β = -0.35, $CI_{95\%}$ = [-0.47, -0.22], t_{232} = -5.46, left-tailed P < 0.001; own impact: β = -0.21, $CI_{95\%}$ = [-0.34, -0.09], t_{232} = -3.29, left-tailed P < 0.001).

Supplementary Note 8: Demographic variable effects in the regressions

We tested the ability of the demographic and general function variables (age, sex, race, IQ and psychiatric disorder) to explain the self-report factors. These variables were entered in the same OLS regressions to estimate their conditional effects, along with the other controls and the affiliation and power averages (one regression per self-report factor). We corrected the p-values for 5 comparisons within each regression, using Bonferroni's method.

Age was significantly negatively correlated with Social Avoidance (β = -0.19, $CI_{95\%}$ = [-0.30, -0.08], t_{810} = -3.28, P_{FWER} = 0.005) and Mood symptoms (β = -0.16, $CI_{95\%}$ = [-0.28, -0.05], t_{810} = -2.78, P_{FWER} = 0.028), but not with Compulsive symptoms (β = -0.12, $CI_{95\%}$ = [-0.24, -0.01], t_{810} = -2.06, P_{FWER} = 0.198). The other variables had no significant associations with any of the factors: sex (Social Avoidance: β = 0.03, $CI_{95\%}$ = [-0.10, 0.17], t_{810} = 0.49, P_{FWER} = 1; Mood: β = -0.04, $CI_{95\%}$ = [-0.18, 0.09], t_{810} = -0.60, P_{FWER} = 1; Compulsive: β = 0.11, $CI_{95\%}$ = [-0.02, 0.25], t_{810} = 1.64, P_{FWER} = 0.503), race (Social Avoidance: β = 0.05, $CI_{95\%}$ = [-0.11, 0.21], t_{810} = 0.57, P_{FWER} = 1; Mood: β = 0.05, $CI_{95\%}$ = [-0.11, 0.21], t_{810} = 0.59, P = 1; Compulsive: β = 0.09, $CI_{95\%}$ = [-0.07, 0.26], t_{810} = 1.09, P_{FWER} = 1), IQ score (Social Avoidance: β = 0.02, $CI_{95\%}$ = [-0.05, 0.09], t_{810} = 0.50, P_{FWER} = 1; Mood: β = -0.01, $CI_{95\%}$ = [-0.08, 0.06], t t_{810} = -0.29, P_{FWER} = 1; Compulsive: β = -

0.08, $CI_{95\%}$ = [-0.15, -0.01], t_{810} = -2.33, P_{FWER} = 0.099), and current psychiatric diagnosis (Social Avoidance: β = 0.29, $CI_{95\%}$ = [-0.06, 0.64], t_{810} = 1.62, P = 0.526; Mood: β = 0.36, $CI_{95\%}$ = [0.00, 0.71], t_{810} = 1.99, P_{FWER} = 0.237; Compulsive: β = 0.32, $CI_{95\%}$ = [-0.04, 0.67], t_{810} = 1.75, P_{FWER} = 0.406).

These age effects suggest competing hypotheses that can be tested in subsequent work. For example, the younger and older participants who engage in online research may be different: e.g., older adults who participate in online studies are likely more comfortable with technology than their peers, which may filter out high-symptom older adults more so than high-symptom younger adults. Age itself may also be a filter: for example, high-symptom individuals may increasingly lose function over the years, reducing the probability they engage in online research. Other classes of hypotheses relate to cohort effects: maybe younger people are more likely to report symptoms (e.g., because reduced stigma around mental health).

Supplementary Note 9: There are no significant behavioral or self-report factor effects related to timepoint during COVID-19 pandemic

We carried out this study during the COVID-19 pandemic. COVID-19 related effects (e.g., physical social distancing) could have affected our results. We assessed the pandemic's impact using the timing of our samples relative to COVID-19. Most of the initial sample data was collected before the FDA granted full approval for a COVID-19 vaccine on August 23, 2021, a significant milestone in the pandemic. Most participants were collected either four months before (all in the Initial sample), or after this date (some in the Initial and the rest in the Validation sample). We created a binary variable on this vaccine approval date to contrast two new vaccine-related subsamples and compared their behavior (average affiliation and power locations) and self-report factor scores (Social Avoidance, Mood and Compulsion) using regressions. There were no

statistically significant effects in behavior (affiliation: β = -0.04, $CI_{95\%}$ = [-0.19, 0.11], t_{811} = -0.49, P = 0.63); power: β = -0.05, $CI_{95\%}$ = [-0.2, 0.1], t_{811} = -0.61, P = 0.541) or self-report factors (Social Avoidance: β = -0.05, $CI_{95\%}$ = [-0.21, 0.1], t_{811} = -0.71, P = 0.48; Mood: β = -0.1, $CI_{95\%}$ = [-0.25, 0.05], t_{811} = -1.33, P = 0.18; Compulsion: β = 0.01, $CI_{95\%}$ = [-0.14, 0.16], t_{811} = -0.16, P = 0.87).
