# Supplementary material for: Social avoidance can be quantified as navigation in abstract social space
Source: Commun Psychol. 2025 Mar 25;3:51. doi: 10.1038/s44271-025-00215-8 (PMC11936828; doi:10.1038/s44271-025-00215-8)
Supplement: Supplementary file 3 — Reporting summary [file 44271_2025_215_MOESM3_ESM.pdf]

nature portfolio

Corresponding author(s): Matthew Schafer
Daniela Schiller

Last updated by author(s): Jan 30, 2025

Reporting Summary

Nature Portfolio wishes to improve the reproducibility of the work that we publish. This form provides structure for consistency and transparency in reporting. For further information on Nature Portfolio policies, see our Editorial Policies and the Editorial Policy Checklist.

Statistics

For all statistical analyses, confirm that the following items are present in the figure legend, table legend, main text, or Methods section.

n/a	Confirmed	
☐	☒	The exact sample size (*n*) for each experimental group/condition, given as a discrete number and unit of measurement
☐	☒	A statement on whether measurements were taken from distinct samples or whether the same sample was measured repeatedly
☐	☒	The statistical test(s) used AND whether they are one- or two-sided<br>*Only common tests should be described solely by name; describe more complex techniques in the Methods section.*
☐	☒	A description of all covariates tested
☐	☒	A description of any assumptions or corrections, such as tests of normality and adjustment for multiple comparisons
☐	☒	A full description of the statistical parameters including central tendency (e.g. means) or other basic estimates (e.g. regression coefficient) AND variation (e.g. standard deviation) or associated estimates of uncertainty (e.g. confidence intervals)
☐	☒	For null hypothesis testing, the test statistic (e.g. F, t, r) with confidence intervals, effect sizes, degrees of freedom and P value noted<br>*Give P values as exact values whenever suitable.*
☒	☐	For Bayesian analysis, information on the choice of priors and Markov chain Monte Carlo settings
☐	☒	For hierarchical and complex designs, identification of the appropriate level for tests and full reporting of outcomes
☐	☒	Estimates of effect sizes (e.g. Cohen's *d*, Pearson's *r*), indicating how they were calculated

Our web collection on statistics for biologists contains articles on many of the points above.

Software and code

Policy information about availability of computer code

Data collection	Combination of custom javascript, html and css, with experiment hosting on Pavlovia
Data analysis	Python 3, with a combination of custom code and open source packages (e.g., Pandas, scipy); more details in github repo: github.com/matty-gee/social_avoidance

For manuscripts utilizing custom algorithms or software that are central to the research but not yet described in published literature, software must be made available to editors and reviewers. We strongly encourage code deposition in a community repository (e.g. GitHub). See the Nature Portfolio guidelines for submitting code & software for further information.

Data

Policy information about availability of data

All manuscripts must include a data availability statement. This statement should provide the following information, where applicable:

- Accession codes, unique identifiers, or web links for publicly available datasets
- A description of any restrictions on data availability
- For clinical datasets or third party data, please ensure that the statement adheres to our policy

Data is available at osf.io/yvts6

Research involving human participants, their data, or biological material

Policy information about studies with human participants or human data. See also policy information about sex, gender (identity/presentation), and sexual orientation and race, ethnicity and racism.

Reporting on sex and gender	Sex was determined by self-report. Sex was included as a covariate in analyses and was not found to statistically relate to any of the behavioral measures in the study.
Reporting on race, ethnicity, or other socially relevant groupings	Participant race was collected via self-report and included as a control variable in analyses. It was not found to statistically relate to any of the behavioral measures in the study.
Population characteristics	There were two samples. The first sample was on average 38.4 years old, 49.7% female and 75.3% white. The second sample was on average 33.2 years old, 58% female and 79.2% white.
Recruitment	Participants were recruited online through the online platform Prolific. There
Ethics oversight	The IRB at Mount Sinai School of Medicine approved the protocol.

Note that full information on the approval of the study protocol must also be provided in the manuscript.

Field-specific reporting

Please select the one below that is the best fit for your research. If you are not sure, read the appropriate sections before making your selection.

☐ Life sciences ☒ Behavioural & social sciences ☐ Ecological, evolutionary & environmental sciences

For a reference copy of the document with all sections, see nature.com/documents/nr-reporting-summary-flat.pdf

Behavioural & social sciences study design

All studies must disclose on these points even when the disclosure is negative.

Study description	This is an online sample of participants completing a choose-your-own-adventure game, along with self-report questionnaires. The data are quantitative.
Research sample	There were two samples, both recruited online via Prolific. The first sample was on average 38.4 years old, 49.7% female and 75.3% white. The second sample was on average 33.2 years old, 58% female and 79.2% white. The samples are representative of America broadly.
Sampling strategy	Two samples were recruited. The first sample overlapped with a previous study and thus its sample size was determined by how many subjects returned. The second sample size was determined by power analysis, and was a random sample of participants on Prolific.
Data collection	Data collection happened online.
Timing	The first sample was collected from April 2021 to January 2022. The second sample was collected in a 4-day period in February 2022.
Data exclusions	Subjects were included based on pre-determined criteria: post-task memory performance, plausible average reaction times (within +/- 2 standard deviations of mean) and successfully completed attention checks during post-task questionnaires. The first sample included 579/733 participants and the second sample included 255/296 participants.
Non-participation	No participants dropped out or declined participation.
Randomization	There was no randomization.

Reporting for specific materials, systems and methods

We require information from authors about some types of materials, experimental systems and methods used in many studies. Here, indicate whether each material, system or method listed is relevant to your study. If you are not sure if a list item applies to your research, read the appropriate section before selecting a response.

Materials & experimental systems

n/a	Involved in the study
☒	☐ Antibodies
☒	☐ Eukaryotic cell lines
☒	☐ Palaeontology and archaeology
☒	☐ Animals and other organisms
☒	☐ Clinical data
☒	☐ Dual use research of concern
☒	☐ Plants

Methods

n/a	Involved in the study
☒	☐ ChIP-seq
☒	☐ Flow cytometry
☒	☐ MRI-based neuroimaging

Plants

Seed stocks: n/a

Novel plant genotypes: n/a

Authentication: n/a
